# Supply of basic necessities to vulnerable populations during the COVID-19 pandemic: Empirical evidence from Shanghai, China

**DOI:** 10.3389/fpubh.2022.1008180

**Published:** 2022-10-26

**Authors:** Qian Wang, Ruiming Dai, Tiantian Zhang, Jiaru Li, Tao Sheng, Bin Wu

**Affiliations:** ^1^Fudan Institute on Ageing, Fudan University, Shanghai, China; ^2^Center for Population and Development Policy Studies, Fudan University, Shanghai, China; ^3^School of Public Health, Fudan University, Shanghai, China; ^4^Shanghai Haiyul Information Technology Co. Ltd., Shanghai, China; ^5^School of Computer Science and Technology, Fudan University, Shanghai, China

**Keywords:** emergency material allocation, China, COVID-19, vulnerable populations, community engagement, economic crisis

## Abstract

**Background:**

In spite of initial widespread skepticism, city lockdown has been proved to be an effective short-term tool in containing and delaying the spread of a viral epidemic. The measures to ensure the supply of the basic necessities adequately and equitably, especially for those vulnerable ones has become a major challenge faced by all countries taking a city lockdown measure during the epidemic.

**Methods:**

Data was collected through relevant government documents, work records, policy reports, media reports and the online-work information platform designed by the research group. Based on these references, the study analyzed the mainly technical difficulties and the countermeasures of the supply process, and summarized the key characteristics of basic necessities supply strategy for vulnerable groups in Shanghai.

**Results:**

The supply strategy for vulnerable groups in Shanghai covers 16 districts, 232 streets and 6,028 neighborhood communities, which has already been in test running in April in some districts. The practical experience in Shanghai solved three key materials supply problems (lack of purchase channels, insufficient material reserves, insufficient transportation capacity) faced by government during the city lockdown, and showed three essential characteristics (overall coordination, community-centered intervention, technical support).

**Conclusions:**

The findings in this study may provide some suggestions to other countries about how to better manage the preparation, dispatch and transportation of basic necessities in shortage for those vulnerable ones during the city lockdown.

## Introduction

In late February 2022, a major outbreak of SARS-CoV-2 infection spread rapidly throughout Shanghai, China (1). The viral gene sequencing results of those positive cases indicated that most of them were infected with the Omicron BA 2.2 variant, a sub lineage of the omicron variant of SARS-CoV-2. The Omicron variant had demonstrated an increased transmissibility and immune escape capability relative to other strains (2–5). Together with the progressive waning of the protection against the infection associated with previous infections or vaccination (6–11), these characteristics led to large Omicron epidemics in Shanghai. According to the Shanghai Municipal Health Commission, from February 26 to June 29, a total of 58,137 symptomatic infections and 591,518 asymptomatic infections were reported (12).

Although the BA.2 subvariant was proved to be the least pathogenic among all existing strains of SARS-CoV-2, a higher rate of mortality was reported in unvaccinated people, especially those older adults, which had been confirmed in Hongkong (13). In shanghai, the vaccination rate of the older adults has remained low (about 62% of 5.8 million people older than 60 have been vaccinated, and only 38% have received a booster vaccination). If strict public health measures were not taken, once the epidemic spreads completely, the number of severe cases and deaths among those unvaccinated older adults could be high. For these reasons, the strict pandemic control strategies were taken in Shanghai during the Omicron epidemic, including city lockdown, large scale viral nucleic acid and antigen screening, quarantine, isolation of infected persons and close contacts, etc.

However, restriction on social activities and mobility brought inconvenience to local citizens, resulting in a severe shortage of basic necessities such as food, daily necessities, epidemic prevention materials and drugs. The lockdown policy showed two main impacts on people's materials access: (1) on the demand side, the closure of the city led to panic buying by residents, resulting in a sharp increase of demand in a short time (14); (2) while on the supply side, conventional retail outlets and supermarkets were forced to close, logistics were disrupted and commodity prices rose sharply.

Besides, during the period of city lockdown, the channels for individuals to independently go to supermarkets were blocked, and intermediary based food purchasing such as group purchase became the most important or even the only channel for citizens to obtain all kinds of basic living necessities. Group purchase is an intermediary food access method based on takeout, which requires buyers to submit the list of shopping needs online through WeChat groups, WeChat mini programs or other Apps, which has been introduced in previous research (15). However, online group purchasing is not accessible to non-netizens populations, which might lead to a serious material shortage of them. The 49th statistical report on the development of China's Internet released by China Internet Network Information Center (CNNIC) showed that older adults aged 60 and above were the main group of non-netizens, which accounted for 39.4% of the total non-netizens by December 2021 (16). For those vulnerable populations such as older adults who cannot access the internet and the disabled, group purchasing online is almost impossible. Therefore, in order to prevent some serious survival and safety problems such as hunger and disease caused by the shortage of basic necessities, it's important for the government to find out the solution about how to ensure the supply of basic necessities for those vulnerable populations during the city lockdown.

The current research subjects have made good explorations on the allocation model of emergency resource and the optimization methodology of emergency logistics. For example, some studies established multi-objective programming model and stochastic programming model to optimize the medical supplies distribution from the perspective of minimize the distribution time, logistics cost (17, 18) mortality (19), the number of rescue point (20) and so on (21). Other studies discussed on the optimization approach of emergency logistics network for the perspective of supply chain, distribution chain and the whole logistics network. However, these studies mostly focused on the reasonable distribution of insufficient materials such as epidemic prevention materials and medicines during the epidemic. Few studies have attempted to discuss how to guarantee the basic necessities supply in the face of the lack of purchase channels of conventional materials caused by urban blockade.

In order to ensure the supply of basic necessities for vulnerable groups during the city lockdown, the research group cooperated with Shanghai Municipal Commission of Commerce, the Civil Affairs Bureau and other government departments to design corresponding solutions according to the material supply problems encountered by residents, and developed an online-work information platform to improve the efficiency and information transparency of material supply. This study mainly focuses on two perspectives for the supply of basic necessities: (1) the social welfare security of vulnerable people based on communities; (2) the operation mode of the basic living material supply system under the extreme scenario of complete urban blockade. The study aims to introduce the basic necessities supply strategy among vulnerable populations during the omicron epidemic in Shanghai, in order to provide reference for other countries and regions to better deal with the shortage of basic necessities of vulnerable groups during the city lockdown.

## Methods

### Data source

#### Work record

Data for the basic necessities supply strategy in Shanghai was collected through relevant government documents, work records, policy reports and media reports.

#### Online-work information platform

We obtained the daily demand and supply data of basic necessities for vulnerable groups from the online information platform named “Shanghai vulnerable populations' basic necessities supply management information platform,” which was jointly developed by Commission of Commerce, the Civil Affairs Bureau and our research group, including about the web side and mobile app side. The mobile app was used by residents or community committees to collect the basic information and material demand information of vulnerable populations, and the web side was used by Shanghai Municipal Commission of Commerce to deal with these information and allocate materials. The platform was freely accessible for all the permitted users (the commerce committee, vulnerable populations, community committees, and research team) from 1st to 30th April (https://sup.singlewindow.sh.cn/manage/).

### Ethics

This study was exempted from the need for ethics approval because the study protocol had neither an intervention nor a breach of privacy or anonymity. All the data used in this study was de-identified.

### Data analysis

The online information platform calculate and statistically analyze the daily demand and supply data automatically from the following aspects:

(1) The reporting condition of all communities, including the number of neighborhood committees reported this round and the number of messages left by neighborhood committees this round.
(2) The demand condition of all communities, including the number of vulnerable groups reported this round, the number of material demands reported this round and the cumulative number of reported material demands.
(3) The processing condition of material demand, including the number of material demands processed this round and the cumulative number of processed material demands.
(4) The written off condition of material demand (after the demand is solved and confirmed by the neighborhood committee, it is regarded as written off), including the number of material demand written off this round and the cumulative number of written off material demands.

## Results

### The overview supply scheme in Shanghai

**Figure 1:**
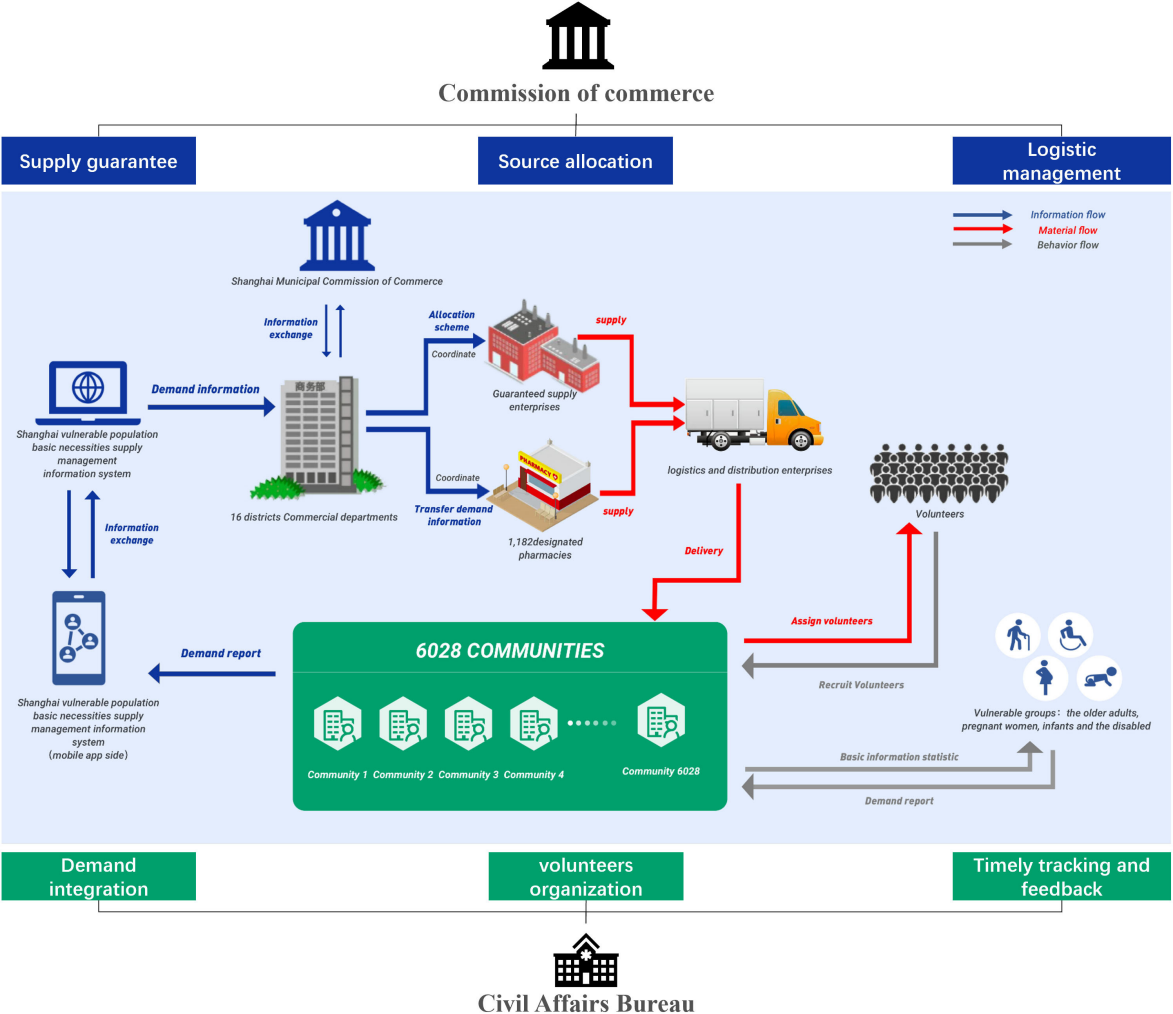
The overview supply scheme of basic necessities for vulnerable populations in Shanghai during the Omicron epidemic.

The overview supply scheme of basic necessities for vulnerable populations in Shanghai during the Omicron epidemic is clearly depicted in Figure 1. In order to deal with the material shortage of those vulnerable populations including older adults, pregnant women, infants, and the disabled, the government implemented the supply scheme by the means of “placing orders by community, allocating orders by Commission of Commerce, distributing orders by supermarkets, and sending orders by community volunteers.” The core processes of the supply scheme included the following steps: (1) the neighborhood committees collected the demand information of those vulnerable populations and formed the demand information database of the whole community; (2) the neighborhood committee reviewed and summarized the demand information, and submitted the total demand of the whole community the Commerce Committee; (3) the commerce commission calculated the daily supply capacity according to the inventory, the emergency purchase quantity and the social donations quantity from other regions, and allocated the materials according to the demand of each community; (4) the Commerce Commission transferred the material demand information to supermarkets, wholesale markets, distribution centers, retail pharmacies and other cooperative supply guarantee enterprises to arrange logistics distribution; (5) after the basic necessities were delivered to the community, the neighborhood committee recruited and organized volunteers to send materials to the doorstep of residents; (6) the neighborhood committees reflected the problems of material supplies through the message board of the information platform, and the Commerce Commission should actively deal with the problems fed back by the neighborhood committees; (7) the Commerce Committee counted the quantity of allocated and unallocated materials, and analyzed the demand processed proportion of each community.

### Key problems and countermeasures of basic necessities supply for vulnerable people in Shanghai

#### Solution to problem one: Lack of purchase channels

##### Demand collection by neighborhood committee

**Figure 2:**
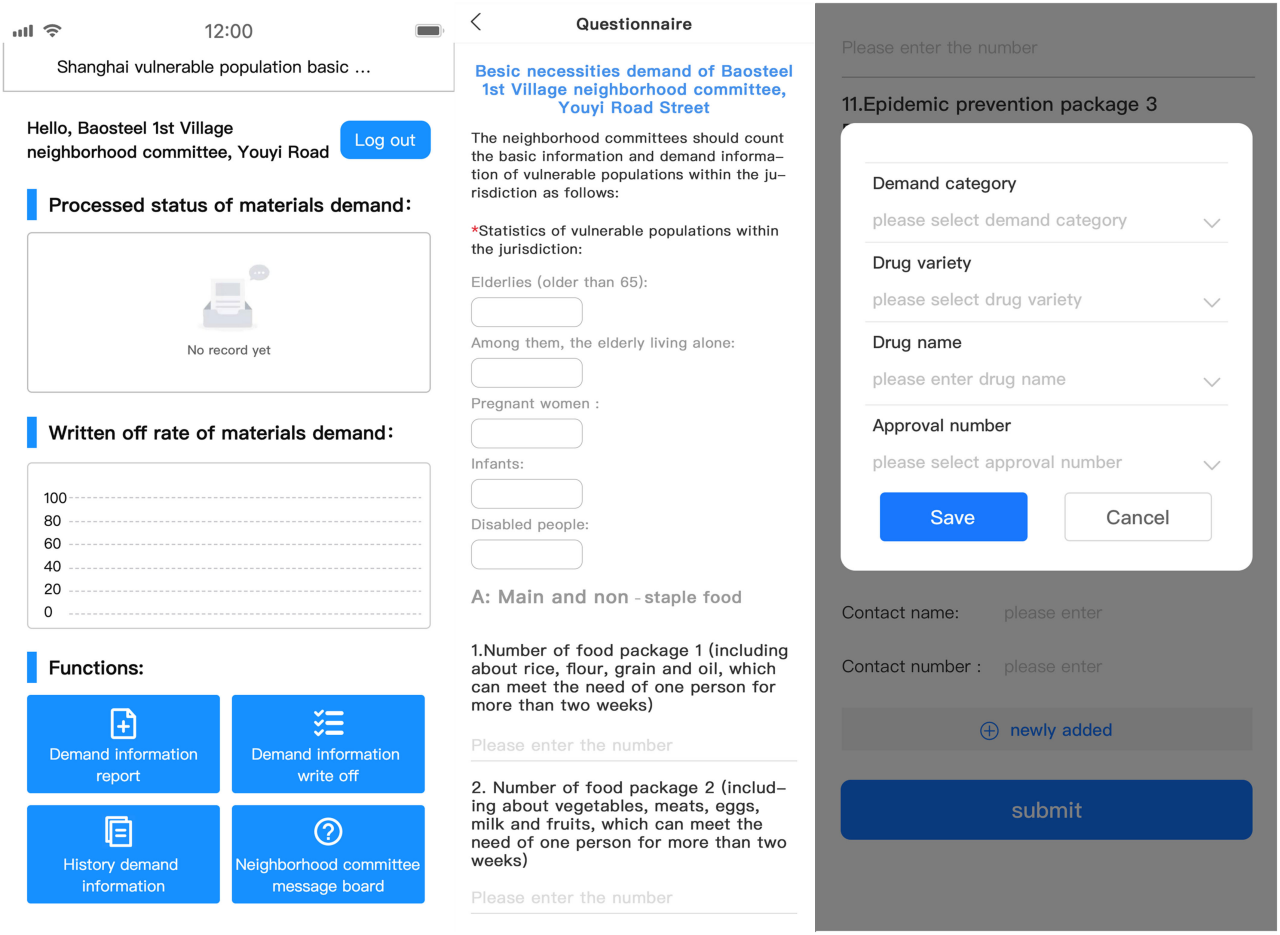
Illustration of the demand collection interface used by communities (mobile app side).

As grassroots organizations in the city, the community committees fully exerted the social capillary role. Taking each community as the basic supply units, the demand of basic necessities of vulnerable populations was counted by four categories: food, daily necessities, epidemic prevention materials, and drugs. Among them, the first three categories were available in the form of packages, including various types of materials for each category. As for drugs, the detailed information such as name, dosage form, product batch, mass, contact name, contact number and other information should be provided accurately. The demand information could be acquired by two main ways (Figure 2): (1) the neighborhood committee took the initiative to inquiry each household with vulnerable individuals to figure out the demand of them; (2) the vulnerable individuals filled in the demand questionnaire issued by the neighborhood committee (Table 1).

**Table 1:** The difficulties and countermeasures of the Shanghai Solution.

**Difficulties**	**Shanghai Solution**	
	**Sub-objective**	**Countermeasures**
Lack of purchase channels	Accurately identify the demand of vulnerable populations	Demand collection by neighborhood committee
	Establish centralized supply channel for vulnerable groups	Placing orders collectively by the units of community
Insufficient material reserves	Ensure the stable source of material supply	Cooperate with guaranteed supply enterprises and designated pharmacies
Insufficient transportation capacity	Complete the “last 100 meters” home delivery	Community volunteers deliver goods to home
	Ensure the timely supply of materials	Timely tracking and feedback of material supply

##### Placing orders collectively by the units of community

After collecting the material demand information, the staffs of the neighborhood committees summed up the total demand of all vulnerable groups of the whole community by categories and placed “centralized orders” by the units of community through the online-work information platform.

#### Solution to problem two: Insufficient material reserves

##### Cooperate with guaranteed supply enterprises and designated pharmacies

The Commerce Commission authorized supermarkets, wholesale markets, distribution centers, e-commerce warehouses, retail pharmacies and other enterprises that strictly implemented epidemic prevention measures to operate normally as guaranteed supply enterprises. The enterprises' employees would be allowed to enter and exit locked-down districts and their home neighborhoods as long as they hold relevant work and other permits, have a negative virus test within 48 h of their activities, and a green health code clearance. After receiving the demand information of vulnerable groups, the Commerce Commission allocated the materials according to the demand amount and the daily supply capacity. As for drugs, the medication demand information of vulnerable groups should be transferred to designated pharmacies according to geographical location, from where the older adults with chronic disease could get medicine for a long term.

#### Insufficient transportation capacity

##### Community volunteers deliver goods to home

Affected by the blockade policy, logistics personnel could not enter the community, while residents could not go out, and the “last 100 meters” from the community gate to the residents' home became a main difficulty for materials transportation. To solve this problem, the neighborhood committees recruited a large number of volunteers from the residents and completed the “last 100 meters” delivery. In order to reduce the gathering, volunteers of each building would take away the materials from the temporary goods stacking area and deliver them door-to-door.

##### Timely tracking and feedback of material supply

To ensure the timely supply of materials, the neighborhood committees could supervise the process of materials supplies through the online-work information platform. In addition, if there were any other supply difficulties in the community, it could be fed back through the “problem reporting” module of the online platform, and the relevant government departments need to deal with the information timely after receiving it.

### The key characteristics of the Shanghai Solution

The Shanghai Solution mainly have the following characteristics, which make it applicable for precise allocation of emergency materials during any epidemic (Table 2).

**Table 2:** Four key characteristics and essential functions of the Shanghai Solution.

**Key characteristics**	**Objective**	**Specific meaning**
Overall coordination	To ensure the smooth coordination of all organizations and the standardized and orderly distribution of materials.	Supported by the Shanghai Municipal Commission of Commerce and the Shanghai Civil Affairs Bureau, the working team of material allocation covered 16 districts, 232 streets and 6,028 neighborhood communities, which also established close cooperative relations with some supply enterprises and designated pharmacies.
Community-centered intervention	To understand, convey and satisfy vulnerable groups' needs timely and quickly.	The main process of demand information collection, placing orders collectively, receiving and distributing materials should be completed by communities.
Technical support	To ensure the transparency and traceability of material allocation.	All the supply process could be monitored visually, real-time and dynamically.

#### Overall coordination

The working team of the distribution of basic necessities was headed by the Shanghai Municipal Commission of Commerce and the Shanghai Civil Affairs Bureau, under the leadership of the mayor of Shanghai municipal government in charge of this sector, together with other municipal departments concerned. During the Omicron epidemic, the team established close cooperative relations with some supply enterprises and designated pharmacies to ensure the sufficient storage of emergency materials. The 16 district-level commercial departments were responsible for guiding and arranging the materials allocations according to the demand of communities within the jurisdiction, and submitting the demand information to cooperative enterprises and designated pharmacies for distribution, and completing the precise allocation of emergency materials. The Shanghai Civil Affairs Bureau took the responsibility of ensuring the timely communication between 6,031 neighborhood committees (in 232 streets) and citizens, and took the lead in coordinating the work among the municipal and district-level civil affairs departments.

#### Community-centered intervention

The implementation of the basic necessities supply program for vulnerable groups in Shanghai was carried out by the units of the community. As the grass-roots main body of the infiltration of state administrative power (22, 23), the community have the functions of management, service, guarantee of residents' rights, education and maintenance of social stability, so as to reduce the pressure of the government during the urban blockade. On the one hand, the community can connect upper- and lower-level organizations in the process of governance (24), transferring vulnerable groups' needs upward, and organizing and coordinating residents and volunteers to effectively implement the government's material distribution plan. On the other hand, the community is also a place where residents live and have emotional interactions with each other (25). This character enable the community to understand people's needs timely, guarantee social equality during a crisis and help protect the rights of vulnerable groups.

#### Technical support

**Figure 3:**
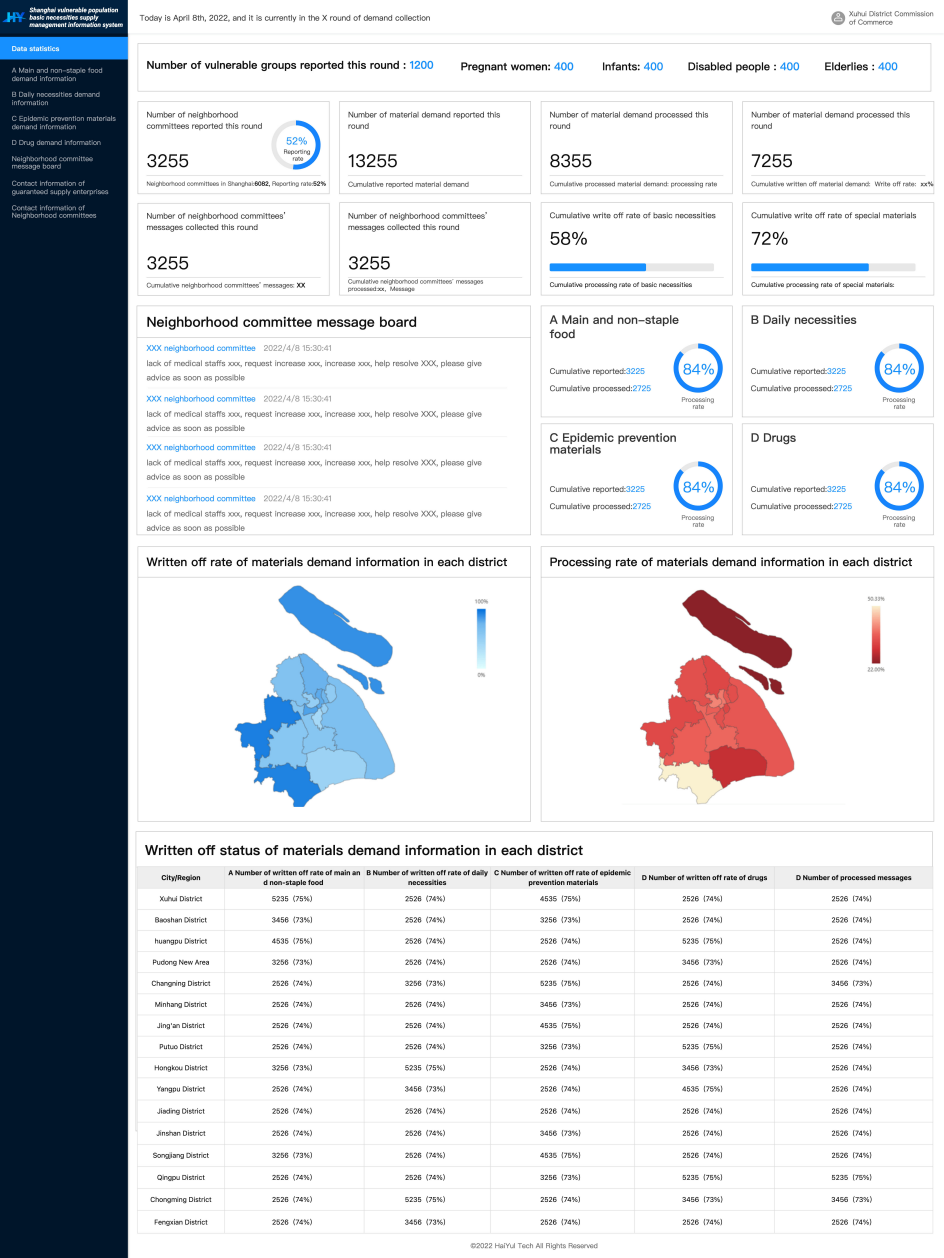
Illustration of the system homepage (part of the dashboard showing demand and distribution data) used by the government.

**Figure 4:**
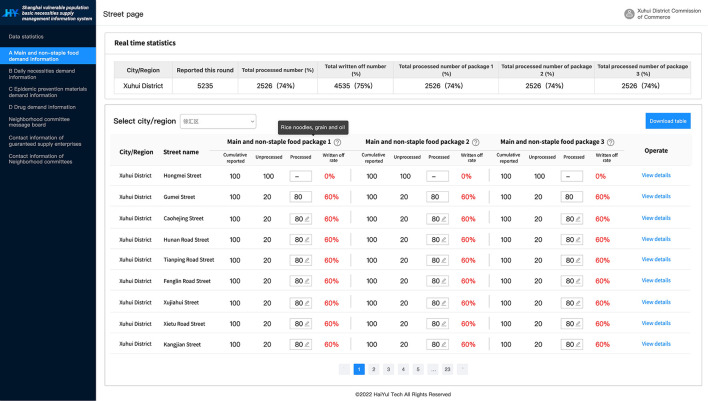
Illustration of the materials allocation interface used by the commission of commerce (web side).

The Shanghai Solution explored to establish a supportive online-work information platform named “Shanghai vulnerable populations' basic necessities supply management information platform,” including the web side and mobile app side. The platform was constructed with the following functions (Figures 3, 4): (1) Collection of daily demand data for each community; (2) Visually, real-time and dynamically monitoring of each district's demand, allocated material amount and cumulative allocation to demand ratio; (3) Providing neighborhood committee with problem feedback board.

### The practical effect of the Shanghai Solution

Form April 1st to 16th, the solution for the supply of basic necessities for vulnerable groups began trial operation in Shanghai. Until 16th April, the program had already provided basic necessities supply services for about 13,000 vulnerable people. During the trial operation period, a total of about 18,800 sets of materials were provided for vulnerable groups (including 17,700 sets of food packages, 260 sets of daily necessities packages, 751 sets of epidemic prevention items and 78 drugs), meeting 100% of the submitted materials needs. The Shanghai Solution guaranteed that all the vulnerable populations could have stable channel to obtain basic necessities, which protect those vulnerable ones from suffering food security and medical shortage problems caused by the urban blockade. Furthermore, with the support of online platform, all data were reviewed through logical calculation to relieve the pressure of government's staffs, reduce data errors, and achieve the real-time monitoring of the material distribution process, which could greatly improve the accuracy and efficiency of material supply.

## Discussion

In this study, we systematically introduced the solution adopted to solve the basic necessities supply problem of vulnerable groups during the Omicron epidemic in Shanghai. Our study indicated that the Shanghai Solution had the following advantages: (1) Accurately meet the material needs of vulnerable groups. Through the combination of community door-to-door investigation and residents' self-reporting, the material demand of vulnerable groups were counted, and the demand information would submit to the supply side. (2) Achieve the transparency and traceability of the material distribution process. The online platform displayed the information of demand, the distribution progress and the quantity of allocated materials to relevant staffs of the neighborhood committee and the commerce commission. Timely supervision and interference were made to avoid the mistakes in the distribution process. (3) Combine automation and intelligent computing to improve the efficiency and fairness of material distribution. The relevant departments uploaded the daily material demand and distribution information, and the system automatically calculated the demand and allocated quantity of each community, on which the allocation plan was based. (4) Avoid crowd gathering. The form of centralized procurement by community and volunteers' delivery goods to home avoids the gathering of people.

During the Omicron epidemic, Shanghai experienced a more severe shortage of materials than in previous outbreaks in other cities. However, the most urgent problem in Shanghai was not the lack of stock in warehouses and supermarkets, but the poor purchase channels. On the one hand, since many staff were blocked at home, there was a severe shortage of manpower in major suppliers and logistics companies, which made it difficult to transport a large number of goods (26, 27). On the other hand, residents had limited procurement channels and could only obtain materials through group purchase, which might be difficult to meet specific needs for vulnerable groups, such as drugs for older adults with chronic diseases, assistive devices for the disabled and pregnant women and infants' supplies, etc. (28). Therefore, Shanghai Solution focused on the vulnerable groups, accurately identified the needs of vulnerable groups with material shortages during the urban blockade, and successfully solved the “last kilometer (i.e., from supermarkets and other suppliers to communities)” and the “last 100 meters (i.e., from the community's material stacking point to residents' homes)” problem in logistics, which effectively ensured the materials supply of vulnerable groups, and avoided the basic survival problems and social stability caused by the shortage of food, drugs and epidemic prevention materials.

Previous studies about the allocation of materials usually focused on the solution of a technical problem in the process of distribution (29, 30). For example, a study used fuzzy number and credibility theory to establish a model to find the optimal order quantity of emergency supplies under the epidemic situation (31). Another study proposed a method to select a short-term transregional material coordination plan to solve the short-term material supply problem and a system dynamics model to simulate the long-term evolutionary state of COVID-19 and the subsequent material requirements (32). Compared with these studies, we aimed to introduce the whole process of the basic necessities supply strategy target for vulnerable groups, which include information collection, demand statistics, place orders collectively, allocation calculation, material allocation and verification, material receiving and distribution. After a month of trial operation, the basic necessities supply of vulnerable groups was ensured during the most stringent period of the urban blockade, which might provide reference for other countries and regions in the distribution strategy of emergency supplies such as food and daily necessities, drugs, medical materials, etc.

In the supply scheme of basic necessities for vulnerable people in Shanghai, community played an important role as the main body of grass-roots governance. On the one hand, community engagement can be critical for creating local and context-specific interventions (33). Through this “bottom-up approach,” communities participate in “decision-making processes of planning, design, governance and delivery of services aimed at improving population health and reducing health inequalities” (34). On the other hand, community engagement is conductive to building interpersonal trust, which could be benefit to protect people's mental health during the lockdown and even decrease the possible suicide risks after the lockdown. Previous studies showed that fears mostly pertained to “containment measures” (isolation, loneliness) both in lockdown and post-lockdown, and the “Interpersonal Trust” theory emerged as a protective factor in post-lockdown for dealing with suicide risk (35, 36).

There are several limitations in this study. First, the evaluation on the effect of the implementation of the plan is not sufficient. This study mainly focuses on the introduction of material supply solution for vulnerable groups in Shanghai. Due to the epidemic, the solution's practical effect and the satisfaction of vulnerable groups have not been investigated and revisited. The follow-up study will supplement the revisit survey for vulnerable groups to evaluate the practical effects of the program. Second, the data and information collected in this study are mainly from work records of government staffs. As a retrospective study, there may be some subjective problems of recall bias and description. In addition, the research focuses on the macro perspective of Shanghai Solutions, so it is almost impossible to explain the specific solutions at each step in detail.

## Conclusion

In spite of initial widespread skepticism, city lockdown has been proved to be an effective short-term tool in containing and delaying the spread of a viral epidemic, which can buy time for governments to mobilize an effective response in order to better prepare. Correspondingly, how to ensure the supply of the basic necessities adequately and equitably, especially for those vulnerable ones has become a major challenge for all countries around the world. In this study, we introduced a citywide strategy of basic necessities supply among vulnerable groups during the omicron epidemic in Shanghai. The findings in this study may provide some suggestions to other countries about how to better manage the preparation, dispatch and transportation of basic necessities in shortage during the city lockdown.

## Data availability statement

The raw data supporting the conclusions of this article will be made available by the authors, without undue reservation.

## Author contributions

All authors made substantial contributions to conception and design, acquisition of data, or analysis and interpretation of data, took part in drafting the article or revising it critically for important intellectual content, agreed to submit to the current journal, gave final approval of the version to be published, and agree to be accountable for all aspects of the work.

## Funding

This study was supported by the National Natural Science Foundation of China (Grant Number: 71874033) and Key Project of Philosophy and Social Science Research of the Ministry of Education (Grant Number: 20JZD027).

## Conflict of interest

Authors JL and BW were employed by Shanghai Haiyul Information Technology Co. Ltd. The remaining authors declare that the research was conducted in the absence of any commercial or financial relationships that could be construed as a potential conflict of interest.

## Publisher's note

All claims expressed in this article are solely those of the authors and do not necessarily represent those of their affiliated organizations, or those of the publisher, the editors and the reviewers. Any product that may be evaluated in this article, or claim that may be made by its manufacturer, is not guaranteed or endorsed by the publisher.

## References

[1] Zhang X Zhang W Chen S. Shanghai's life-saving efforts against the current omicron wave of the COVID-19 pandemic. Lancet. (2022) 399:2011–2. 10.1016/S0140-6736(22)00838-8 35533708 PMC9075855

[2] Arora P Zhang L Rocha C Sidarovich A Kempf A Schulz S. Comparable neutralisation evasion of SARS-CoV-2 omicron subvariants BA1, BA2, and BA3. Lancet Infect Dis. (2022) 22:766–7. 10.1016/S1473-3099(22)00224-9 35427493 PMC9005119

[3] Chen Z Deng X Fang L Sun K Wu Y Che T. Epidemiological characteristics and transmission dynamics of the outbreak caused by the SARS-CoV-2 Omicron variant in Shanghai, China: a descriptive study. medRxiv. (2022) 29:100592. 10.1101/2022.06.11.22276273 36090701 PMC9448412

[4] Viana R Moyo S Amoako DG Tegally H Scheepers C Althaus CL. Rapid epidemic expansion of the SARS-CoV-2 omicron variant in southern Africa. Nature. (2022) 603:679–86. 10.1038/s41586-022-04411-y 35042229 PMC8942855

[5] Pearson CAB Silal SP Li MWZ Dushoff J Bolker BM Abbott S. Bounding the levels of transmissibility and immune evasion of the Omicron variant in South Africa. medRxiv. (2021). 10.1101/2021.12.19.21268038

[6] Levin EG Lustig Y Cohen C Fluss R Indenbaum V Amit S. Waning immune humoral response to BNT162b2 Covid-19 vaccine over 6 months. N Engl J Med. (2021) 385:e84. 10.1056/NEJMoa2114583 34614326 PMC8522797

[7] Xin Q Wu Q Chen X Han B Chu K Song Y. Six-month follow-up of a booster dose of CoronaVac in two single-centre phase 2 clinical trials. Nat Commun. (2022) 13:3100. 10.1038/s41467-022-30864-w 35660738 PMC9166693

[8] Notarte KI Guerrero-Arguero I Velasco JV Ver AT de Oliveira MHS Catahay JA. Characterization of the significant decline in humoral immune response six months post-SARS-CoV-2 mRNA vaccination: a systematic review. J Med Virol. (2022) 94:2939–61. 10.1002/jmv.27688 35229324 PMC9088566

[9] Dan JM Mateus J Kato Y Hastie KM Yu ED Faliti CE. Immunological memory to SARS-CoV-2 assessed for up to 8 months after infection. Science. (2021) 371:eabf4063. 10.1126/science.abf4063 33408181 PMC7919858

[10] Collier AY Yu J McMahan K Liu J Chandrashekar A Maron JS. Differential kinetics of immune responses elicited by Covid-19 vaccines. N Engl J Med. (2021) 385:2010–2. 10.1056/NEJMc2115596 34648703 PMC8531985

[11] Wheatley AK Juno JA Wang JJ Selva KJ Reynaldi A Tan H-X. Evolution of immune responses to SARS-CoV-2 in mild-moderate COVID-19. Nat Commun. (2021) 12:1162. 10.1038/s41467-021-21444-5 33608522 PMC7896046

[12] Commission. SMH. The latest statistical table of epidemic data in Shanghai.

[13] Hong Kong Centre for Health Protection of the Department of Health atHA. Statistics on 5th wave of COVID-19. (2022) (accessed May 4, 2022).

[14] Hobbs JE. Food supply chains during the COVID-19 pandemic. Can J Agric Econ. (2020) 68:171–6. 10.1111/cjag.1223734908599

[15] Zhang Y Yang K Hou S Zhong T Crush J. Factors determining household-level food insecurity during COVID-19 epidemic: a case of Wuhan, China. Food Nutr Res. (2021) 65:5501. 33776620 10.29219/fnr.v65.5501 PMC7955523

[16] CINIC. The 49th Statistical Report on China's Internet Development. (2022). Available online at: https://www.cnnic.com.cn/IDR/ReportDownloads/202204/P020220424336135612575.pdf (accessed October 15, 2022).

[17] Wang Y Peng SG Xu M. Emergency logistics network design based on space–time resource configuration. Knowl Based Syst. (2021) 223:107041. 10.1016/j.knosys.2021.107041

[18] Wang HY Wang XP. Optimal material distribution decisions based on epidemic diffusion rule and stochastic latent period for emergency rescue. Int J Math Operation Res. (2009) 1:76–96. 10.1504/IJMOR.2009.022876

[19] Xiang YS Zhuang J. A medical resource allocation model for serving emergency victims with deteriorating health conditions. Ann Operat Res. (2016) 236:177–96. 10.1007/s10479-014-1716-1

[20] Yang B Li XL Du B. [Solving multi-objective emergency scheduling problem in fuzzy environments]. J Syst Manag. (2013) 4:518–25. 10.3969/j.issn.1005-2542.2013.04.012

[21] He YX Liu N. Methodology of emergency medical logistics for public health emergencies. Transp Res Part E Logist Transp Rev. (2015) 79:178–200. 10.1016/j.tre.2015.04.007 32288598 PMC7147567

[22] Ahrends AHPM Ziegler AD Fox JM Chen HF Su YF Xu JC. Current trends of rubber plantation expansion may threaten biodiversity and livelihoods. Glob Environ Chang-Hum Policy Dimens. (2015) 34:48–58. 10.1016/j.gloenvcha.2015.06.002

[23] Zou Y Wang Q Deng M Wang Y. Community intervention system: COVID-19 control in inner mongolia autonomous region, China. Int J Environ Res Public Health. (2021) 18:12857. 10.3390/ijerph182312857 34886579 PMC8657202

[24] Vignoles VLJZ Taylor F Ntontis E. Harnessing shared identities to mobilize resilient responses to the COVID-19 pandemic. Political Psychol. (2021) 42:817–26. 10.1111/pops.12726 33821062 PMC8013210

[25] Parra DCGLF Sarmiento OL Buchner D Brownson R Schimd T Gomez V. Perceived and objective neighborhood environment attributes and health related quality of life among the elderly in Bogota, Colombia. Soc Sci Med. (2010) 70:1070–6. 10.1016/j.socscimed.2009.12.024 20138418

[26] CBN. “Resumption” of the Supply Chain of Basic Livelihood Materials During the Epidemic (2022).

[27] Zhang Jiayi LS Lingjun W Shaohui H. Analysis on the logistics scheme of guaranteed supply under the closure and control of the epidemic in Shanghai. Logist Procure China. (2022) 10:58–60. 10.16079/j.cnki.issn1671-6663.2022.10.030

[28] Chenyan L. Shanghai has built a special channel for emergency special needs for people with special needs. Wen Wei Po. (2022).

[29] Liu J GL Jiang J Jiang D Wang P. Emergency material allocation with time-varying supply-demand based on dynamic optimization method for river chemical spills. Environ Sci Pollut Res Int. (2018) 25:17343–53. 10.1007/s11356-018-1489-1 29654459

[30] Du L. Medical emergency resource allocation model in large-scale emergencies based on artificial intelligence: algorithm development. JMIR Med Inform. (2020) 8:e19202. 10.2196/19202 32584262 PMC7381036

[31] R ZC. Fuzzy random emergency supplies in stock during an outbreak. J Chongqing Univ Commer Indust. (2021) 38:79–85. 10.16055/j.issn.1672-058X.2021.0003.011

[32] Sun H Xu W Yu Y Cai G. An intelligent mechanism for COVID-19 emergency resource coordination and follow-up response. Comput Intell Neurosci. (2022) 2022:2005188. 10.1155/2022/2005188 35747718 PMC9210128

[33] Gilmore B Ndejjo R Tchetchia A de Claro V Mago E Diallo AA. Community engagement for COVID-19 prevention and control: a rapid evidence synthesis. BMJ Glob Health. (2020) 5:e003188. 10.1136/bmjgh-2020-003188 33051285 PMC7554411

[34] Barker KM Ling E Fallah M VanDeBogert B Kodl Y Macauley RJ. Community engagement for health system resilience: evidence from Liberia's Ebola epidemic. Health Policy Plan. (2020) 35:416–23. 10.1093/heapol/czz174 32040166

[35] Costanza A Amerio A Aguglia A Serafini G Amore M Macchiarulo E. From “The Interpersonal Theory of Suicide”to “The Interpersonal Trust”: an unexpected and effective resource to mitigate economic crisis-related suicide risk in times of Covid-19? Acta Biomed. (2021) 92:e2021417. 10.23750/abm.v92iS6.12249 34739460 PMC8851025

[36] Costanza A Macheret L Folliet A Amerio A Aguglia A Serafini G. COVID-19 related fears of patients admitted to a psychiatric emergency department during and post-lockdown in Switzerland: preliminary findings to look ahead for tailored preventive mental health strategies. Medicina (Kaunas). (2021) 57:1360. 10.3390/medicina57121360 34946305 PMC8707997

